# Decreased expression of ARHGAP15 promotes the development of colorectal cancer through PTEN/AKT/FOXO1 axis

**DOI:** 10.1038/s41419-018-0707-6

**Published:** 2018-06-04

**Authors:** Shengli Pan, Yingying Deng, Jun Fu, Yuhao Zhang, Zhijin Zhang, Xiaokun Ru, Xianju Qin

**Affiliations:** 1 Division of Gastrointestinal Surgery, Department of General Surgery, Shanghai Eighth People Hospital, Shanghai, 200232 P.R. China; 2 Department of Ophthalmology, Shanghai Eighth People Hospital, Shanghai, 200232 P.R. China

## Abstract

Copious evidence demonstrates the crucial role of Rho GTPase-activating proteins in human malignancies. The downregulation of Rho GTPase-activating protein 15 (ARHGAP15), a Rac1-specific GAP, has been observed in glioma and pancreatic ductal adenocarcinoma. The present study explored the expression in colorectal cancer (CRC) by quantitative real-time PCR and immunohistochemistry analysis. The possible function of ARHGAP15 in CRC was investegated in vitro and in vivo. We found that ARHGAP15 expression was obviously lower in CRC specimens than in normal colonic mucosa. ARHGAP15 expression was significantly correlated with clinical stage, tumor size metastasis, vital status, and overall survival of CRC patients. ARHGAP15 overexpression inhibited cell growth, migration, and invasion of HT29 and RKO cells in vitro, whereas opposite results were observed in ARHGAP15-silenced LoVo cells. Mechanically, we found that PTEN (phosphatase and tensin homology deleted on chromosome 10) signaling pathway was closely correlated with ARHGAP15 expression by Gene set enrichment analysis with The Cancer Genome Atlas CRC data set. Increased PTEN and Forkhead box protein O1 (FOXO1, a downstream transcription factor of AKT), and decreased phosphorylation of AKT were observed in ARHGAP15-overexpressed HT29 and RKO cells. In addition, ARHGAP15 overexpression increased p21, which was responsible for the accelerated cell growth and S phase arrest, but decreased the protein levels of MMP-2 and MMP-9, which were stimuli for cell metastasis. Notably, upregulating PTEN expression, FOXO1 overexpression and interdicting the activation of AKT pathway with MK2206 suppressed the proliferation and the metastatic ability of ARHGAP15-silenced LoVo cells. In addition, FOXO1 overexpression markedly enhanced the expression and the promoter activity of ARHGAP15. Furthermore, ARHGAP15 overexpression significantly decelerated the pace of tumor growth and metastasis in the lung in vivo. In summary, these results suggest that ARHGAP15 might serve as a tumor suppressor during CRC progression and metastasis through PTEN/AKT/FOXO1-signaling pathway.

## Background

Colorectal cancer (CRC) is the third frequently diagnosed malignancy in human and accounts for a staggering amount of cancer-related death next to lung cancer^1,2^. Surgery, radiotherapy, and chemotherapy are conventional treatment options for CRC patients. Notably, the emergence of targeted drugs gives fresh impetus to the therapy of metastatic CRC^3^. Although the incidence and mortality rates have declined in the past decades among the individuals > 50 years old, there is a steady growth trend for CRC in the younger population^4,5^. Therefore, it is still of great significance to dissect the pathogenesis of CRC.

Rho family of GTPases is a subgroup of the Ras superfamily. Copious evidence demonstrates their crucial role in the initiation and progress of malignancies owing to the regulation on multiple biological processes, such as cytoskeleton reorganization, cell motility, and cell cycle progression^6^. The activity of Rho GTPases is regulated by numerous proteins, of which GTPase-activating proteins (GAPs) are principal negative regulators. Through the enhanced intrinsic hydrolysis of GTP, Rho GTPases can be transformed into inactivated GDP-bound state^7^. Rho GTPase-activating protein 15 (ARHGAP15) is a Rac1-specific GAP^8^. Early studies suggest that ARHGAP15 deregulation is implicated in many abnormalities. For example, ARHGAP15 decreased in glioma, which promoted the aggressive phenotypes of glioma cells through the activation of Rac1^9^. Intronic mutation of ARHGAP15 is associated with diverticular disease^10^. In addition, ARHGAP15 was a prognosis-related biomarker for pancreatic ductal adenocarcinoma^11^.

Phosphatase and tensin homology deleted on chromosome 10 (PTEN) is an important tumor suppressor. By converting phosphatidylinositol-3,4,5-trisphosphate to phosphatidylinositol-4,5-bisphosphate, it antagonizes the effect of Phosphatidylinositol-3-kinase (PI3K), which eventually suppresses AKT activation^12^. Moreover, PTEN was found to be downregulated in CRC^13^ and overexpression of it could restrain the growth of CRC cell lines through the inactivation of AKT pathway^14^. Overexpressing AKT drives the CRC cells into a highly proliferative and invasive state^15^. AKT exerts functions through regulating downstream transcription factors, including the forkhead transcription factor superfamily, such as Forkhead box protein O1 (FOXO1), FOXO3a, FOXO4, and FOXO6^25^. FOXO proteins play key roles in diverse biological processes, such as cell differentiation, stress responses, cell cycle progression, cell apoptosis, and glucose metabolism^16^. FOXO subfamily members have been identified as key tumor suppressors through upregulating the cell cycle inhibitors p21Cip1 and p27Kip1, downregulating the cell cycle regulator cyclin D1/2, and subsequently inducing cell cycle arrest^17–20^.

Previous research reveals that ARHGAP15 deregulation is implicated in many abnormalities, yet no study has focused on the effects of ARHGAP15 on CRC. In the present study, we explored the expression and function of ARHGAP15 in CRC. We found that PTEN/AKT/FOXO1 axis was involved in the anti-proliferation and anti-invasion effects of ARHGAP15.

## Results

### ARHGAP15 was downregulated in human CRC tissues

**Fig. 1:**
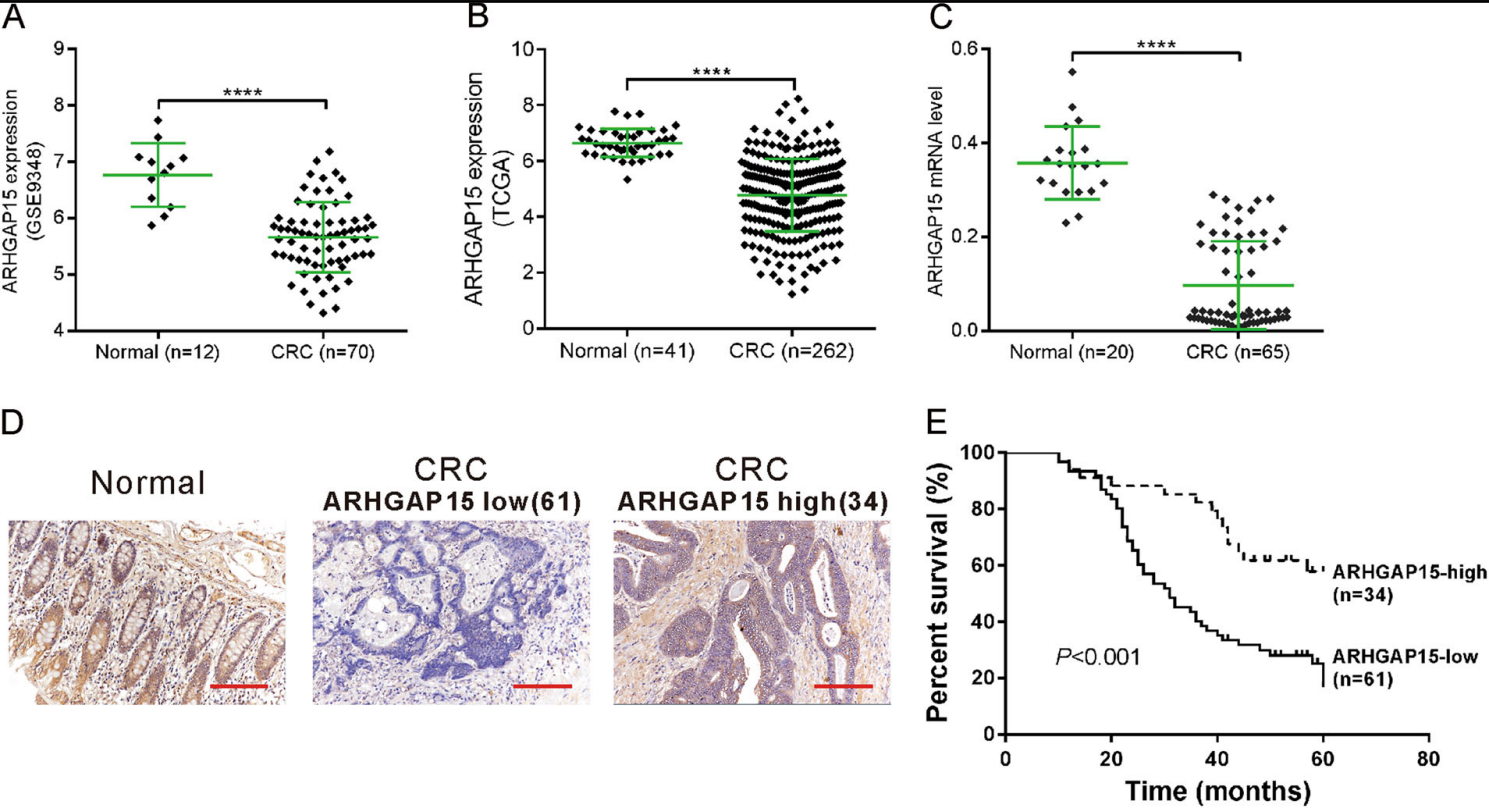
Downregulation of ARHGAP15 in CRC tissues. **a**, **b** ARHGAP15 mRNA expression was analyzed based on two public available data sets: GSE9348 (**a**) and TCGA CRC (**b**). **c** The mRNA levels of ARHGAP15 on our own cohort were determined using qRT-PCR. **d** The protein levels of ARHGAP15 were determined by immunohistochemistry staining. Scale bar: 100 μm. **e** Kaplan–Meier survival curves showing the difference of survival time between patients with low and high expression of ARHGAP15 on CRC samples from 95 patients. ****P < 0.0001

To study ARHGAP15 expression in CRC tissues, two publicly available data sets, GSE9348^26^ downloaded from Gene Expression Omnibus (GEO) database and the CRC data set from The Cancer Genome Atlas (TCGA), were re-analyzed. ARHGAP15 mRNA expression was significantly lower in CRC tissues than in the normal colonic mucosa (*P* < 0.0001, Fig. 1a, b). Further, quantitative real-time PCR (qRT-PCR) analysis performed on our own cohort also indicated the downregulation of ARHGAP15 mRNA expression in CRC tissues (*P* < 0.0001, Fig. 1c).

To investigate the changes of ARHGAP15 protein, we performed Immunohistochemistry (IHC) staining on CRC samples from 95 patients. ARHGAP15 expression was observed in cytoplasm (Fig. 1d). A total of 61 cases showed low expression of ARHGAP15 (64.2%) and 34 cases had high expression of ARHGAP15 (35.8%).

### Downregulated ARHGAP15 correlated with clinicopathologic features of CRC patients

We then analyzed the correlation between ARHGAP15 expression and the clinicopathologic features of CRC. Fisher’s exact test showed that ARHGAP15 expression was significantly correlated with clinical stage (*P* = 0.005), tumor size (*P* = 0.028), metastasis (*P* = 0.020), and vital status (*P* = 0.010) (Table 1). Survival analysis showed that patients with lower ARHGAP15 expression had a shorter survival time than those with higher ARHGAP15 expression (Fig. 1e, *P* < 0.001).

**Table 1:** Association of ARHGAP15 expression with clinicopathological features

Characteristic		*n*	ARHGAP15		*P* value
			High (*n* = 34)	Low (*n* = 61)	
Gender	Male	50	21	29	0.522
	Female	45	13	32	
Age (years)	< 60	49	20	29	0.392
	≥ 60	46	14	32	
Clinical stage	I/II	45	23	22	0.005**
	III	50	11	39	
Tumor size (cm)	< 4.0	38	19	19	0.028*
	≥ 4.0	57	15	42	
Metastasis	Yes	28	5	23	0.020*
	No	67	29	38	
Differentiation	Well/moderate	63	26	37	0.174
	Poor	32	8	24	
Vital status (at followed-up)	Alive	39	20	19	0.010*
	Dead	56	14	42	

Fisher’s exact test was performed. *P < 0.05, **P < 0.01

Subsequently, multivariate Cox regression analysis revealed that tumor size (hazard ratio, 3.07; 95% CI, 1.49–6.31; *P* = 0.002), metastasis (hazard ratio, 3.64; 95% CI, 1.67–7.92; *P* = 0.001) and ARHGAP15 expression (hazard ratio, 3.07; 95% CI, 1.57–6.01; *P* = 0.001) were independent prognostic factors for CRC (Table 2).

**Table 2:** Multivariate Cox regression of prognostic parameters for survival in patients with colorectal cancer

Prognostic parameter	Multivariate analysis		
	HR	95% CI	*P* value
Expression of ARHGAP15 (high vs. low)	3.07	1.57–6.01	0.001**
Clinical stage (I/II vs. III)	1.30	0.75–2.25	0.348
Tumor size ( < 4.0 cm vs. ≥ 4.0 cm)	3.07	1.49–6.31	0.002**
Metastasis (yes vs. no)	3.64	1.67–7.92	0.001**

HR: Hazard ratio; CI: Confidence interval. *P < 0.05, **P < 0.01

### ARHGAP15 influenced the proliferation, cell cycle progression, migration, and invasion of CRC cells

Next, we verified the differential expression of ARHGAP15 in CRC cell lines and normal HIEC by qRT-PCR and western blot. According to Figure S1A and S1B, ARHGAP15 decreased in all of the five CRC cell lines compared with HIEC.

**Fig. 2:**
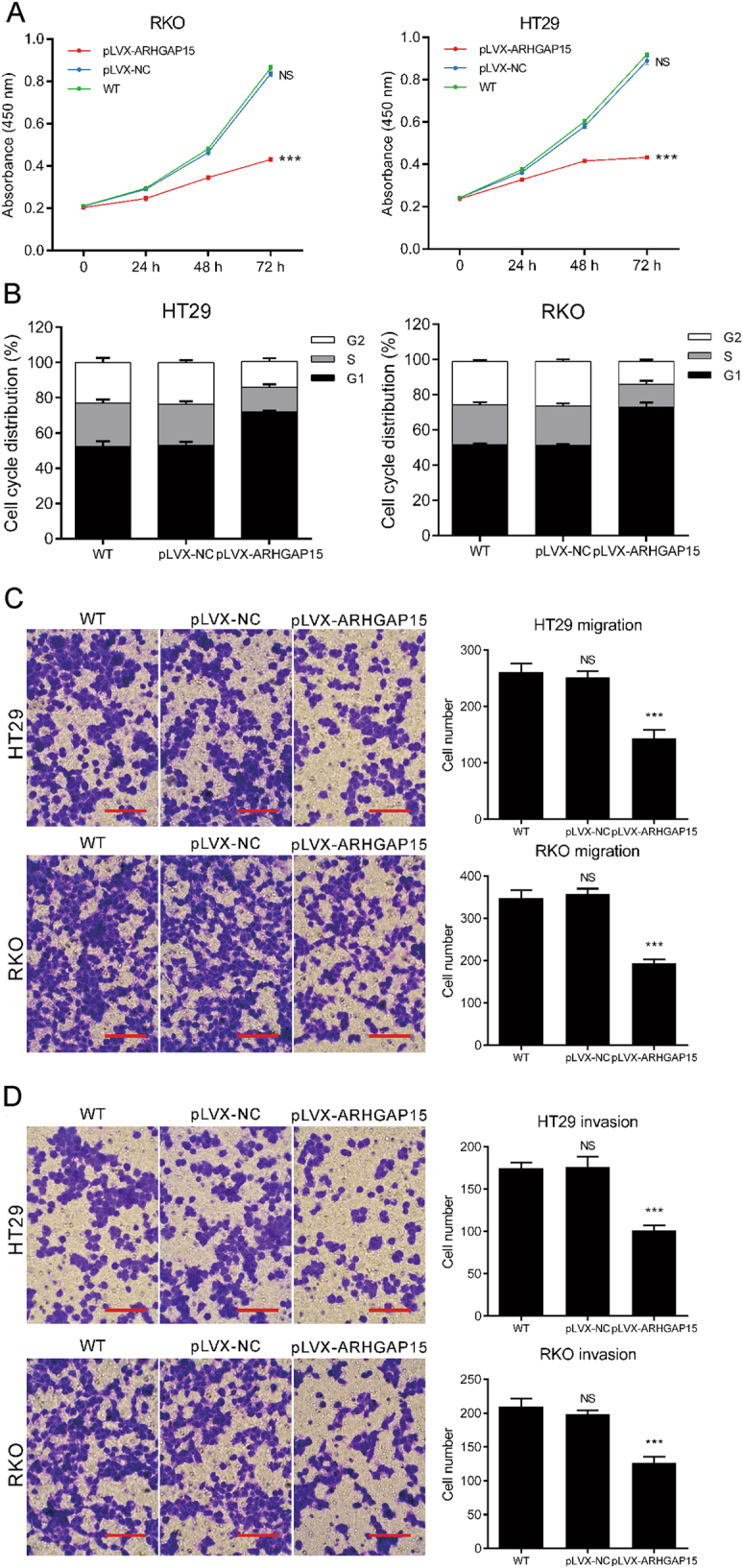
ARHGAP15 overexpression suppressed the proliferation, migration, and invasion and induced G1 phase arrest of CRC cells. HT29 and RKO cells were transfected with pLVX-ARHGAP15 or pLVX-NC. **a** Cell proliferation detected by CCK-8 assay. **b** Cell cycle distribution analyzed by flow cytometry. **c** Cell migration detected by transwell assay. Scale bar: 100 μm. **d** Cell invasion detected by transwell assay. Scale bar: 100 μm. Experiments were repeated three times independently. NS: no significant difference; *** *P* < 0.001

**Fig. 3:**
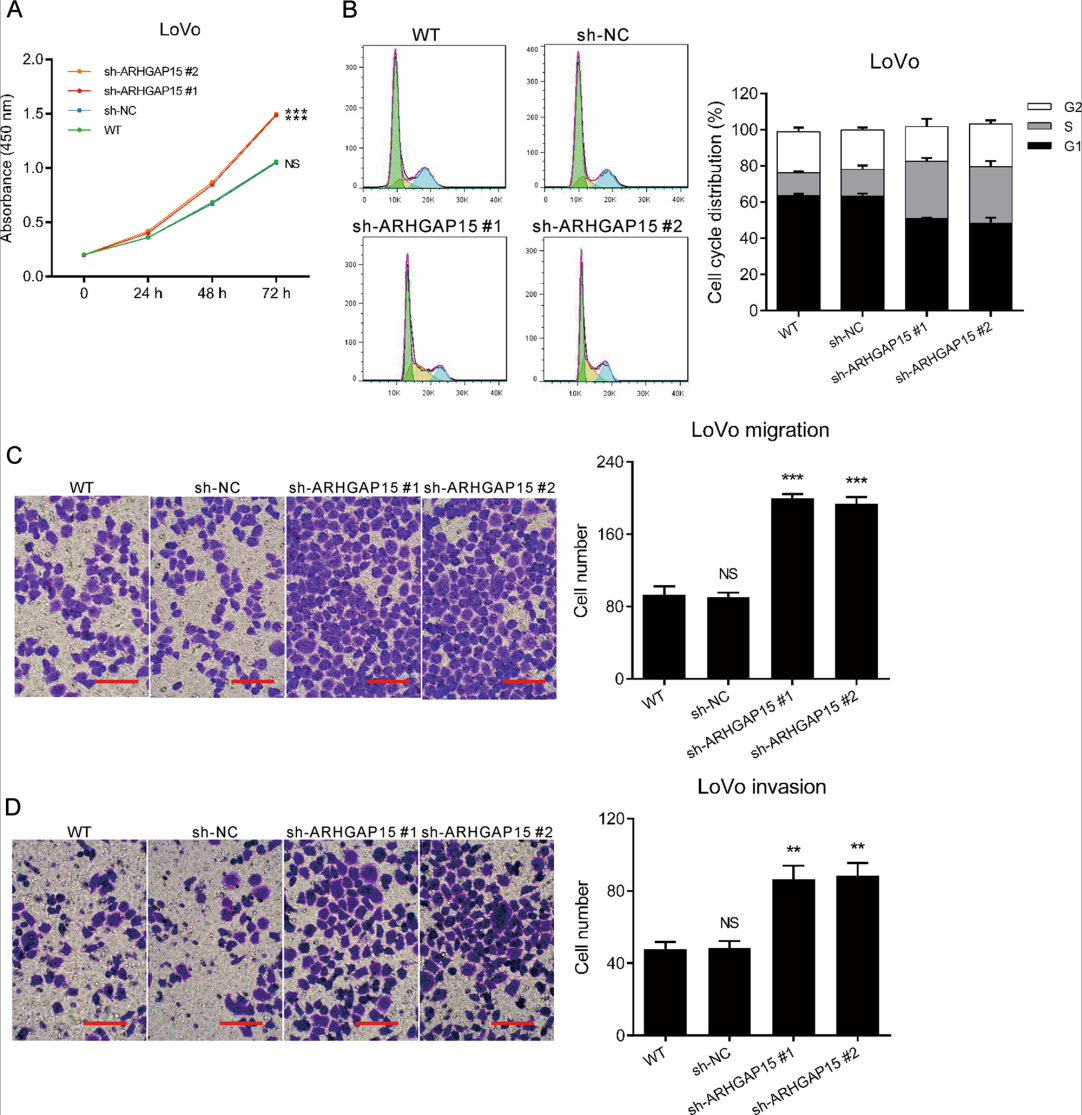
ARHGAP15 silencing facilitated the proliferation, migration, and invasion and induced S phase arrest of CRC cells. LoVo cells were transfected with ARHGAP15 shRNA (sh-ARHGAP15) or control shRNA (sh-NC). **a** Cell proliferation detected by CCK-8 assay. **b** Cell cycle distribution analyzed by flow cytometry. **c** Cell migration detected by transwell assay. Scale bar: 100 μm. **d** Cell invasion detected by transwell assay. Scale bar: 100 μm. Experiments were repeated three times independently. NS: no significant difference; ** *P* < 0.01; *** *P* < 0.001

To investigate the effect of ARHGAP15 on several cellular processes, ARHGAP15 expression was altered in CRC cell lines. Owing to the relative lower expression of ARHGAP15, RKO, and HT29 cells were overexpressed with ARHGAP15 by transducing with pLVX-ARHGAP15, which was confirmed by qRT-PCR (Figure S1C and S1E) and western blot (Figure S1D and S1F). Cell Counting Kit-8 (CCK-8) assay demonstrated the impaired cell proliferation following the overexpression of ARHGAP15 (Fig. 2a). Cell cycle analysis showed the increase of G1 phase cell population (Fig. 2b). Moreover, migrated and invaded cells decreased also (Figs. 2c, d). In conclusion, ARHGAP15 overexpression suppressed the proliferation, migration, and invasion and induced G1 phase arrest of CRC cells. Opposite results were obtained in LoVo cells, a CRC cell line which demonstrated higher ARHGAP15 levels, after knocking down ARHGAP15 expression with specific shRNA. Figures S2A and 2B showed that sh-ARHGAP15 #1 and #2 efficiently reduced ARHGAP15 expression as indicated by qRT-PCR and western blot. The downregulated expression of ARHGAP15 facilitated the proliferation (Fig. 3a), migration (Fig. 3c) and invasion (Fig. 3d) of CRC cells. In addition, we observed more cells were arrested at S phase (Fig. 3b). On balance, ARHGAP15 served as a tumor suppressor to suppress the growth and metastasis of CRC cells.

### ARHGAP15 influenced the development of CRC via modulating PTEN/AKT/FOXO1-signaling pathway

**Fig. 4:**
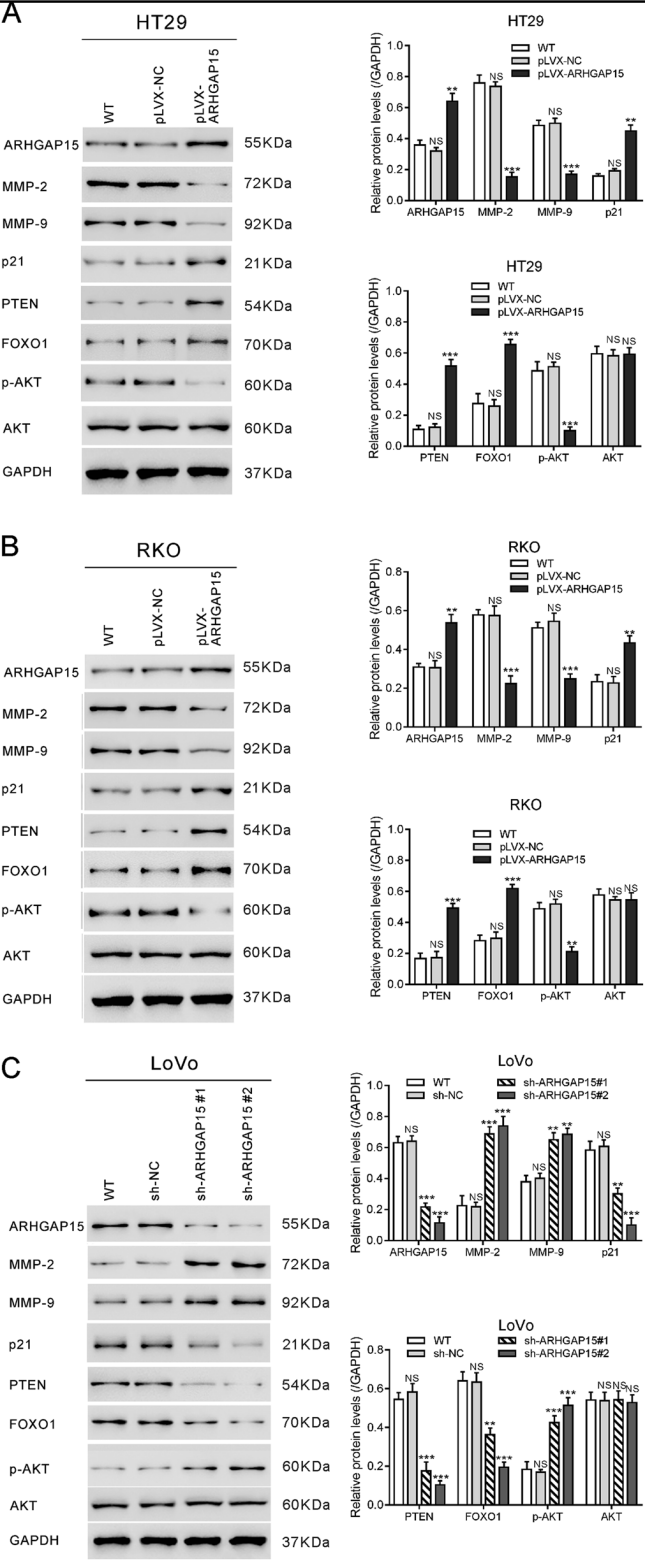
ARHGAP15 modulated PTEN/AKT/FOXO1-signaling pathway. **a**, **b** The protein levels of several pivotal molecules in HT29 (**a**) and RKO (**b**) cells after transfected with pLVX-ARHGAP15 or pLVS-NC. **c** The protein levels of several pivotal molecules in LoVo cells after transfected with ARHGAP15 shRNA (sh-ARHGAP15) or control shRNA (sh-NC). Experiments were repeated three times independently. NS: no significant difference; ** *P* < 0.01; *** *P* < 0.001

Gene Set Enrichment Analysis (GSEA) was then performed using TCGA CRC data set, and PTEN pathway was positively correlated with ARHGAP15 expression in CRC samples (Figure S3). PTEN was found to be downregulated in CRC and overexpression of it could restrain the growth of CRC cell lines through the inactivation of AKT pathway^14^. To examine the effect of ARHGAP15 on AKT pathway, the levels of PTEN, p-AKT, and AKT were assessed by western blotting. The results revealed the increase of PTEN following ARHGAP15 overexpression. Accordingly, phosphorylated AKT at Ser473 decreased in response to the negative regulation of PTEN (Fig. 4a, b). Furthermore, activation of AKT inhibits FOXO1, a member of forkhead family of transcription factors, which is associated with cell proliferation and apoptosis^21,22^. Here, ARHGAP15 overexpression led to an increase in FOXO1 protein expression. In addition, ARHGAP15 overexpression increased p21, which was responsible for the accelerated cell growth and S phase arrest, and downregulated MMP-2 and MMP-9, which were stimuli for cell metastasis. Conversely, ARHGAP15 knockdown brought about absolutely opposite results (Fig. 4c).

**Fig. 5:**
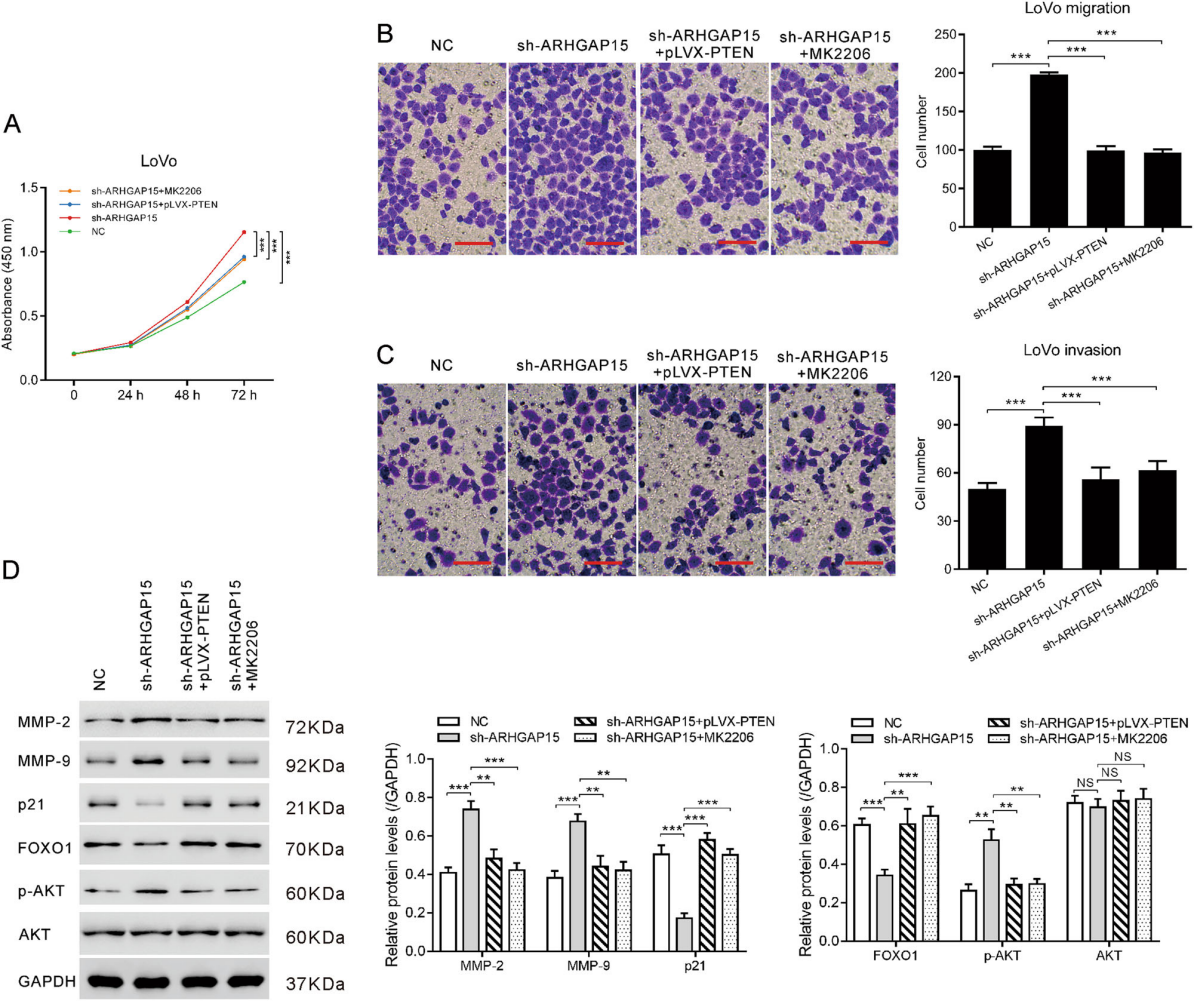
ARHGAP15 influenced the development of CRC via modulating PTEN/AKT/FOXO1-signaling pathway. ARHGAP15-silenced LoVo cells were transfected with pLVX-PTEN or treated with MK2206. Cell proliferation (**a**) was detected by CCK-8 assay. Cell migration (**b**) and invasion (**c**) was detected by transwell assay. Scale bar: 100 μm. Protein levels of related molecules (**d**) was detected by western blot. Experiments were repeated three times independently. NS: no significant difference; **p < 0.01; *** *P* < 0.001

Subsequently, we detected whether PTEN overexpression or AKT inhibitor could abrogate ARHGAP15 silencing enhanced malignant phenotypes. The excessive expression of PTEN was confirmed by qRT-PCR and western blot (Figure S4). CCK-8 assay demonstrated that both upregulating PTEN expression and interdicting the activation of AKT pathway with MK2206 suppressed the viability of ARHGAP15-silenced LoVo cells (Fig. 5a). Similarly, the metastatic ability was impaired also (Figs. 5b, c). Moreover, FOXO1 and p21 increased, whereas MMP-2 and MMP-9 decreased following the inactivation of AKT (Fig. 5d). Taken together, ARHGAP15 exerted biological effect through PTEN/AKT/FOXO1-signaling pathway.

### FOXO1 regulated the expression of ARHGAP15

**Fig. 6:**
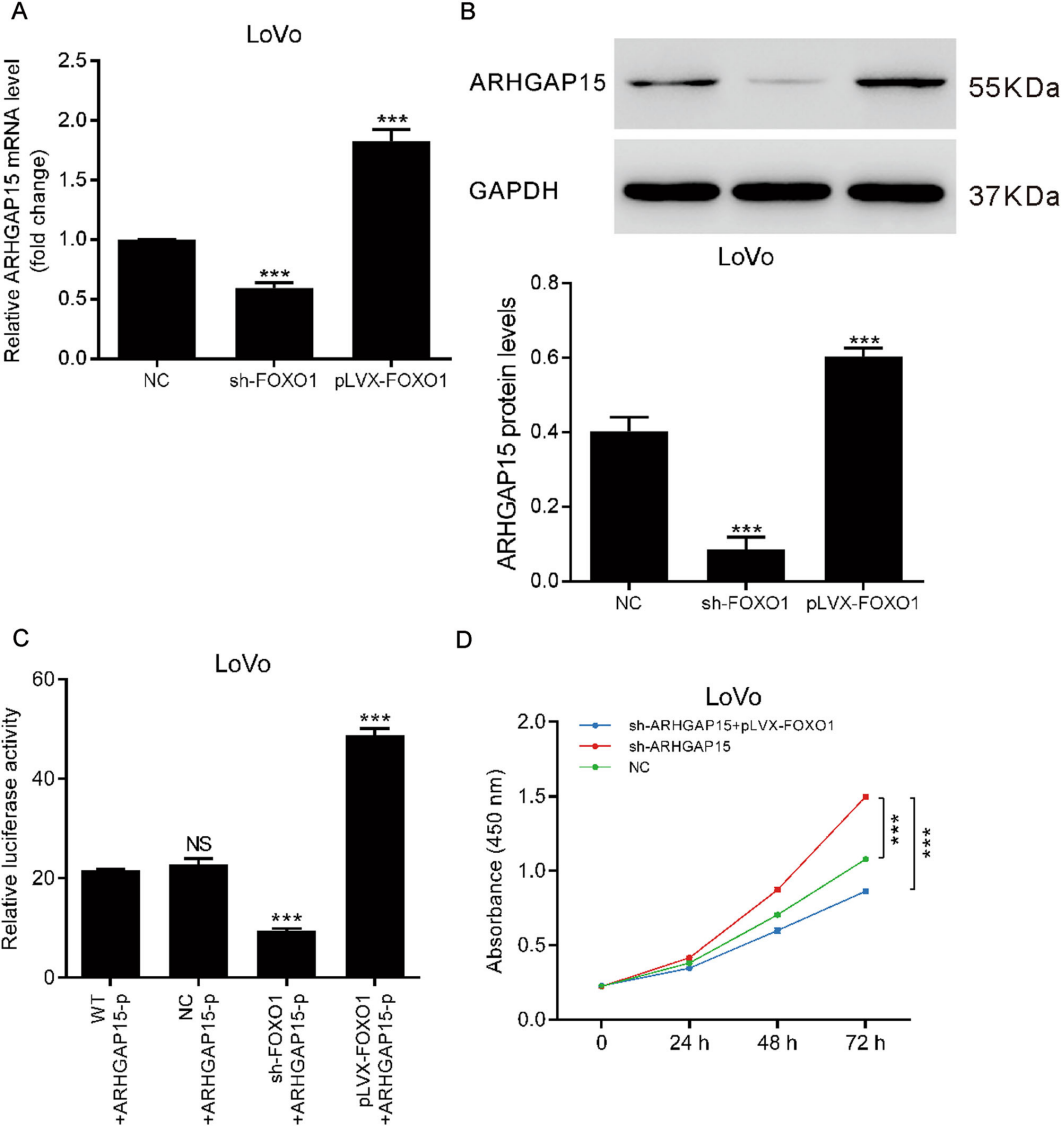
FOXO1 regulated the expression of ARHGAP15. **a**, **b** LoVo cells were transfected with FOXO1 interference or overexpression lentiviral vectors. The regulation of FOXO1 on the mRNA (**a**) and protein (**b**) expression of ARHGAP15 was assessed. **c** ARHGAP15 promoter sequence was cloned into pGL3-basic to construct the luciferase reporter plasmid. After transfected with the recombinant plasmid, luciferase reporter activity was detected. (**d**) Cell viability detected by CCK-8 assay to assess the antagonism between ARHGAP15 silencing and FOXO1 overexpression. Experiments were repeated three times independently. NS: no significant difference; *** *P* < 0.001

As a typical transcription factor, intranuclear FOXO1 modulated the expression of numerous targets associated with multiple biological functions^21^. Hereby, we investigated whether FOXO1 could regulate ARHGAP15 expression. LoVo cells were transfected with lentiviral vectors to alter the expression of FOXO1. Transfection efficiency was ascertained by qRT-PCR and western blot (Figure S5). We could see that upregulated FOXO1 markedly stimulated the expression of ARHGAP15, whereas FOXO1 knockdown repressed ARHGAP15 expression (Fig. 6a, b). Furthermore, a recombinant luciferase reporter plasmid was constructed by cloning the promoter sequence of ARHGAP15 into pGL3-basic and co-transfected into LoVo cells together with FOXO1 interference or overexpression lentiviral vectors. Relative luciferase reporter activity increased with the excessive expression of FOXO1 according to Fig. 6c, indicating that FOXO1 could enhance the promoter activity of ARHGAP15. Notably, FOXO1 overexpression could dramatically suppress cell proliferation elevated by ARHGAP15 knockdown (Fig. 6d). In conclusion, ARHGAP15 and FOXO1 constituted a positive feedback loop, through which they were bounded with each other.

### ARHGAP15 overexpression suppressed the formation and metastasis of CRC in vivo

**Fig. 7:**
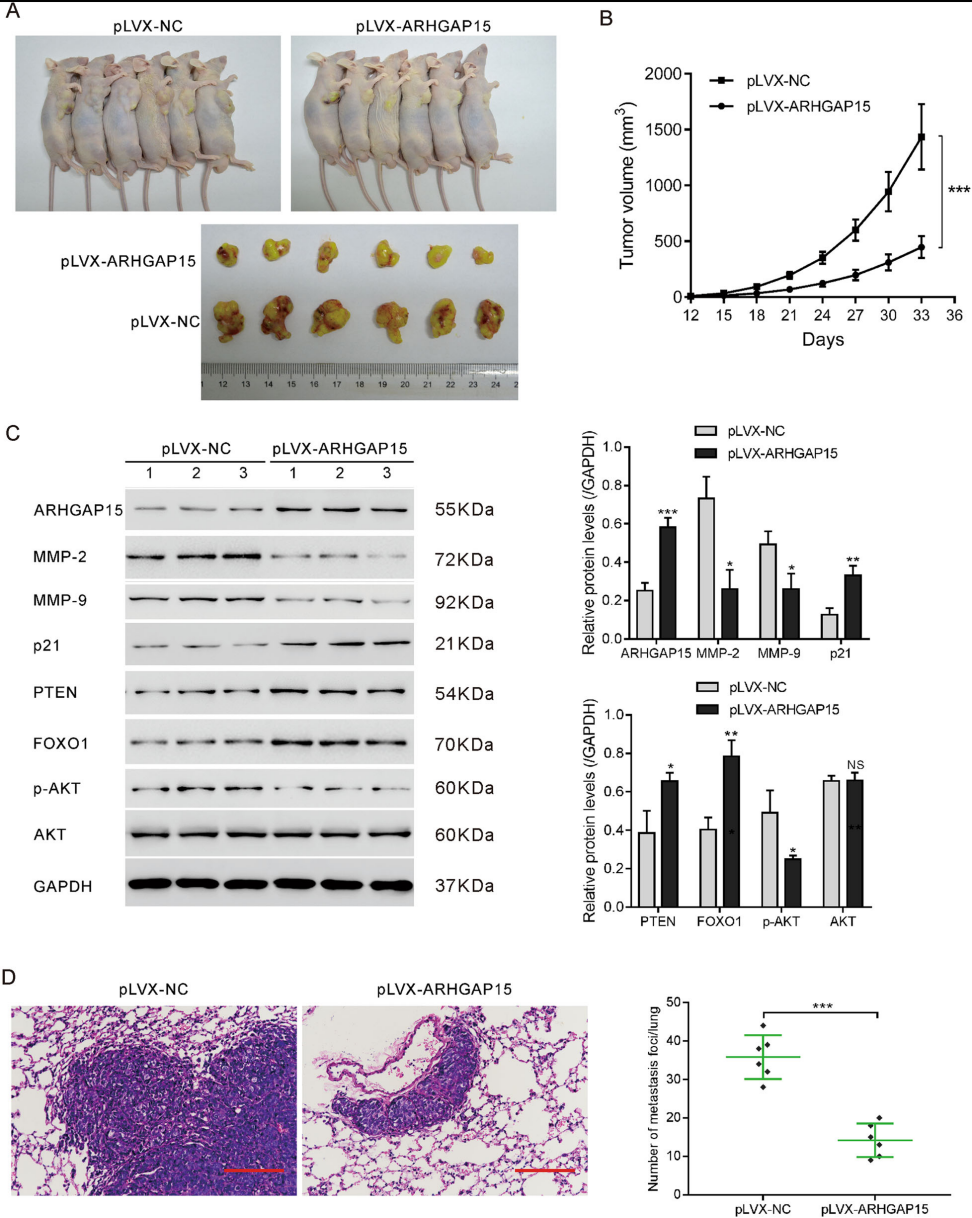
ARHGAP15 hyperexpression suppressed the formation and distant metastasis of CRC in vivo. CRC xenograft mouse model was established to validate the antitumor effect of ARHGAP15 overexpression in vivo. Two groups were divided according to the subcutaneously injection of pLVX-NC or pLVX-ARHGAP15 transfected RKO cells to nude mice (six mice per group). **a** The pictures of the tumor-bearing mice and tumors. **b** Tumor growth curves of the two groups. **c** Western blot analysis of the crucial molecules in the tumors. **d** HE staining of lung metastases. Scale bar: 100 μm. NS: no significant difference; *P < 0.05; **P < 0.01; *** *P* < 0.001

Nude mice were subcutaneously injected with empty vectors or pLVX-ARHGAP15 transduced RKO cells to construct CRC xenograft model. Results demonstrated that tumors grew more slowly in ARHGAP15-overexpressed mice (Fig. 7a, b). Western blot showed the differential expression of PTEN/AKT/FOXO1 pathway members in the tumor tissues of the two groups (Fig. 7c). All the results were in accord with those in vitro. Collectively, ARHGAP15 overexpression significantly decelerated the pace of tumor growth in vivo.

To determine the effects of ARHGAP15 overexpression on metastasis in vivo, in vivo metastasis mouse model was established by tail vein injection of empty vectors or pLVX-ARHGAP15 transduced RKO cells. As shown in Fig. 7d, the number of metastasis foci in the lung of ARHGAP15 overexpression group was decreased to 43.1% of that in the vector group, indicating that metastasis was markedly suppressed by ARHGAP15 overexpression.

## Discussion

ARHGAP15, a Rac1-specific GAP^8^, has been found to be de-regulated in human tumors. For instance, a lower expression of ARHGAP15 was shown to be significantly correlated with a poorer survival of patients with pancreatic ductal adenocarcinoma^11^. ARHGAP15 expression was regulated by Forkhead box P3 in glioma^9^. In this study, we discovered that ARHGAP15 expression was downregulated in CRC tissues (Fig. 1). Consistently with the above results, ARHGAP15 reduced in CRC cell lines (Fig. 2a). In addition, ARHGAP15 expression was significantly correlated with clinical stage, tumor size metastasis, vital status, and overall survival of CRC patients (Fig. 1 and Table 1). Furthermore, multivariate analysis identified that ARHGAP15 protein level may serve as an independent prognostic factor in patients with CRC (Table 2). For validating the anticancer activity of ARHGAP15 in vitro, lentiviral vectors were applied to interfere with or enhance ARHGAP15 expression. As expected, impaired cell proliferation, migration, and invasion as well as a G1 phase arrest were observed following the overexpression of ARHGAP15 (Fig. 2), whereas reverse results were obtained after knocking down ARHGAP15 expression (Fig. 3). In addition, ARHGAP15 overexpression significantly decelerated the pace of tumor growth and metastasis in the lung in vivo (Fig. 7). All of these data indicate that ARHGAP15 acts as a tumor suppressor in CRC.

Activation of the PI3K/AKT pathway has been demonstrated to play a pivotal role in the carcinogenesis and metastasis of various cancers^23^. The impaired expression of PTEN and activation of AKT signaling has been observed in CRC tissues^13^. Overexpression of PTEN could restrain the growth of CRC cells^14^, whereas overexpression of AKT induces the proliferation and invasion of CRC cells^15^. Deleted in liver cancer 1 (DLC1), another Rho GTPase-activating protein, has been reported to interact with PTEN to regulate the migration of breast cancer cells^22^. Here, GSEA with TCGA data set showed that ARHGAP15 expression was correlated with the PTEN signaling pathway in CRC specimens, which promoted us to further explore the association of ARHGAP15 and PTEN/AKT signaling. We found that ARHGAP15 overexpression caused increased expression of PTEN and decreased phosphorylation of AKT at Ser473 (Fig. 4). Thus, we propose that ARHGAP15 may inhibit aggressive phenotypes by regulating PTEN/AKT signaling. We found that PTEN overexpression or AKT inhibitor MK2206 can rescue the promotional influence on cell proliferation, migration, and invasion caused by ARHGAP15 knockdown (Fig. 5). These data suggest that ARHGAP15 may serve as a tumor suppressor for CRC through PTEN/AKT signaling.

Matrix metalloproteinases (MMPs), such as MMP-2 and MMP-9, modulate cell–cell and cell–extracellular matrix interactions via degrading various cell adhesion molecules, thereby playing vital roles in cancer cell migration, invasion, metastasis, and angiogenesis^24^. AKT overexpression in CRC cells could increase the expression of MMP-2 and MMP-9^15^. In the present study, ARHGAP15 overexpression decreased the levels of MMP-2 and MMP-9 (Fig. 4), which may be due to the decreased phosphorylation of AKT. Correlation between ARHGAP15 and MMP-2/9 may explain the increased migration and invasion of CRC cells.

FOXO proteins, which play key roles in cell differentiation, stress responses, cell cycle progression, cell apoptosis, and glucose metabolism^16^, have been suggested to be negatively regulated by AKT^25^. Here, ARHGAP15 overexpression caused increased expression of FOXO1 (Fig. 4). Moreover, FOXO1 overexpression markedly enhanced the expression and the promoter activity of ARHGAP15. In addition, FOXO1 overexpression can rescue the promotional influence on cell proliferation caused by sh-ARHGAP15 (Fig. 6). These results prove that ARHGAP15 affects the growth of CRC cells partially by regulating FOXO1 expression. We suspect that ARHGAP15 and FOXO1 establish a positive feedback loop to inhibit the carcinogenesis of CRC.

## Conclusions

Our study suggest that ARHGAP15 might be a novel prognostic biomarker for CRC. Our data also revealed that ARHGAP15 could inhibit cell proliferation, migration, and invasion of CRC cells through the PTEN/AKT/FOXO1-signaling pathway. These findings may provide new insights into the molecular mechanisms associated with CRC progression and the development of therapeutic strategies for CRC treatment.

## Methods

### Patients and tissue samples

This study has been approved by the ethics committee of Shanghai Eighth People Hospital (Shanghai, China). Ninety-five patients with CRC who underwent a curative surgical procedure between the Jan 2007 and Jan 2011 at Division of Gastrointestinal Surgery, Department of General Surgery, Shanghai Eighth People Hospital were enrolled in this study. Written informed consent was obtained from all patients. Clinicopathological features, such as age, gender, tumor size, clinical stage, tumor differentiation, metastasis, and survival time was retrieved from patient records. The mean age of enrolled patients 56 years (ranging from 35 to 77 years), and 52.6% were men. The samples included 45 cases of clinical stage I and II, and 50 cases of clinical stage III CRC. CRC tissues from all the 95 patients were formalin-fixed and paraffin-embedded, whereas 65 cases of CRC tissues and 20 adjacent normal colonic mucosa were available for qRT-PCR analysis.

### Quantitative real-time PCR

TRIzol Reagent (invitrogen, 15596–026) was used for total RNA isolation from cultured cells or clinical tissues. Complementary DNA was synthesized from RNA using the RevertAid First Strand cDNA Synthesis Kit (Fermentas, K1622). Amplification reaction was performed with Maxima SYBR Green/ROX qPCR Master Mix (Thermo Scientific, K0223) on ABI Prism 7300 Real-Time PCR System (Applied Biosystems, USA). The primers used were as follows. ARHGAP15 (NM_018460.3): forward 5′-AGCACACATTGAATGGGCCAA-3′; reverse 5′-TTGATAGCGTGGAACCAATCC-3′. PTEN (NM_000314.4): forward 5′-TCAGGCGAGGGAGATGAGAG-3′; reverse 5′-CGAAGAGGAGGCGAGAAACG-3′. FOXO1 (NM_002015.3): forward 5′-GGTTAGTGAGCAGGTTAC-3′; reverse 5′-GGCACAGTCCTTATCTAC-3′. GAPDH (NM_001256799.1): forward 5′-CACCCACTCCTCCACCTTTG-3′; reverse 5′-CCACCACCCTGTTGCTGTAG-3′.

### IHC analysis

Formalin-fixed, paraffin-embedded sections were prepared from CRC tissues and normal colonic tissues, and IHC staining was performed with ARHGAP15 antibody (Invitrogen, Carlsbad, CA, USA; PA5-31530) as previously described^9^. The sections were reviewed by two investigators and graded into ARHGAP15-high-expression group (> 20% of tumor cells were positively stained) and ARHGAP15-low-expression group (< 20% of tumor cells were positively stained).

### Cell culture

Normal human intestinal crypt cells (HIEC) and human CRC cell lines, HT29, RKO LoVo, SW620, and SW480, were purchased from the Shanghai Cell Bank, Chinese Academy of Sciences (Shanghai, China). Cells were grown in Dulbecco's Modified Eagle Medium supplemented with 10% fetal bovine serum (FBS) and 1% penicillin–streptomycin. All the cells were incubated in a humidified atmosphere at 37 °C with 5% CO_2_.

### Lentiviral vectors construction and cell transfection

RNA interference sequences targeting specific genes was synthesized and cloned into linearized pLKO.1 plasmids (Addgen, Cambridge, MA, USA). The interference sites and corresponding oligo sequences were as below: ARHGAP15 (NM_018460.3, position 267–285: GCCAAAGTAAATCCATGAT): forward CCGGGCCAAAGTAAATCCATGATCTCGAGATCATGGATTTACTTTGGCTTTTT; reverse AATTAAAAAGCCAAAGTAAATCCATGATCTCGAGATCATGGATTTACTTTGGC. ARHGAP15 (NM_018460.3, position 652–670: CCTTCTACAGTCAGATATT): forward CCGGCCTTCTACAGTCAGATATTCTCGAGAATATCTGACTGTAGAAGGTTTTT; reverse AATTAAAAACCTTCTACAGTCAGATATTCTCGAGAATATCTGACTGTAGAAGG. ARHGAP15 (NM_018460.3, position 1034–1052: CCGTGGTTTGTAAAGCAAT): forward CCGGCCGTGGTTTGTAAAGCAATCTCGAGATTGCTTTACAAACCACGGTTTTT; reverse AATTAAAAACCGTGGTTTGTAAAGCAATCTCGAGATTGCTTTACAAACCACGG. FOXO1 (NM_002015.3, position 1039–1057: CCTACACAGCAAGTTCATT): forward CCGGCCTACACAGCAAGTTCATTCTCGAGAATGAACTTGCTGTGTAGGTTTTT; reverse AATTAAAAACCTACACAGCAAGTTCATTCTCGAGAATGAACTTGCTGTGTAGG. FOXO1 (NM_002015.3, position 1338–1356: GCTCAAATGCTAGTACTAT): forward CCGGGCTCAAATGCTAGTACTATCTCGAGATAGTACTAGCATTTGAGCTTTTT; reverse AATTAAAAAGCTCAAATGCTAGTACTATCTCGAGATAGTACTAGCATTTGAGC. FOXO1 (NM_002015.3, position 1908–1926: GCAGCCAGGCATCTCATAA): forward CCGGGCAGCCAGGCATCTCATAACTCGAGTTATGAGATGCCTGGCTGCTTTTT; reverse AATTAAAAAGCAGCCAGGCATCTCATAACTCGAGTTATGAGATGCCTGGCTGC. DH5a-competent cells were transfected with the recombinant plasmids and cultured in Luria broth agar plates. Positive clones were identified by PCR and gene sequencing. Recombinant plasmids together with the packaging plasmids psPAX2 and pMD2G were co-transfected into 293T cells using lipofectamine 2000 (Invitrogen). Virus solution was collected for cell transfection.

To elevate the expression of ARHGAP15, PTEN and FOXO1, the coding sequences were synthesized using the following primers and cloned into pLVX-puro (Clontech, Palo Alto, CA, USA). ARHGAP15 (NM_018460.3): forward 5′-GCGAATTCATGCAGAAATCTACAAATTCTGATA-3′ (*Eco*RI); reverse 5′-CGGGATCCTCAGTCTTCCTCTGAGCCGAAGATC-3′ (*Bam*HI). PTEN (NM_002141.4): forward 5′-GCGAATTCATGACAGCCATCATCAAAGAGATCG-3′ (*Eco*RI); reverse 5′-CGGGATCCTCAGACTTTTGTAATTTGTGTATGC-3′ (*Bam*HI). FOXO1 (NM_002015.3): forward 5′-GCGAATTCATGGCCGAGGCGCCTCAGGTGGTG-3′ (*Eco*RI); reverse 5′-CGGGATCCTCAGCCTGACACCCAGCTATGTGTC-3′ (*Bam*HI). Underscores denoted the cutting sites of *Eco*RI and *Bam*HI. Lentivirus packaging was performed after sequence identification.

### Western blot analysis

Cultured cells or clinical tissues were lysed with radioimmunoprecipitation assay for protein extraction. Proteins of different molecular weights were separated with sodium dodecyl sulfate–polyacrylamide gel electrophoresis. Protein expression was quantified according to the gray value after normalized to GAPDH. The antibodies used were as below: ARHGAP15 (Invitrogen, PA5-31530), PTEN (Cell Signaling Technology (CST), Danvers, MA, USA; #9552), p21 (CST, #2947), MMP-2 (Abcam, Cambridge, MA, USA; Ab14311), MMP-9 (Abcam, Ab38898), AKT (CST, #9272), p-AKT (CST, #9271), FOXO1 (Abcam, Ab52857), GAPDH (CST, #5174).

### CCK-8 assay

CRC cells were seeded into 96-well plates (5 × 10^3^/well) and incubated overnight. Then the cells were divided into different groups. Different virus suspensions were added to each well for cell transfection. After incubated sequentially for 0, 24, 48, and 72 h, cell counting kit-8 solution (CCK-8, SAB biotech. College Park, MD, USA) was added and absorbance at 450 nm was measured to assess cell viability.

### Cell cycle analysis

CRC cells (5 × 10^5^ per well) were seeded into six-well plates and incubated for 24 h. Lentiviruses were transduced to interfere with or heighten ARHGAP15 expression. Forty-eight hours later, the cells were digested and washed with precooled phosphate-buffered saline (PBS). In total, 300 μL of PBS contained 10% FBS and 700 μL of −20 °C precooled absolute ethyl alcohol were successively added for cell fixation. After incubation at 4 °C for 24 h, cell suspension was centrifuged and washed with precooled PBS again. In total, 100 μL of RNase A (1 mg/mL, Sigma, St. Louis, MO, USA) was added for cell resuspension. After incubation in dark for 30 min, the cells were stained with 400 μL of propidium iodide (50 μg/mL, Sigma) for 10 min. Cell cycle distribution was analyzed with a flow cytometer (BD Biosciences, Franklin Lakes, NJ, USA, Accuri C6).

### Transwell assay

CRC cells were seeded into six-well plates and incubated for 24 h. Then the cells were divided into different groups and treated with different virus suspensions. 48 h later, the cells were digested and resuspended in cell culture medium containing 1% FBS at a density of 2 × 10^5^/mL. For invasion assay, the upper chamber was pre-coated with 1 mg/mL matrigel (BD Biosciences). Subsequently, 0.3 mL cell suspension was added to the upper chamber, whereas 0.7 mL medium supplemented with 10% FBS was added to the lower chamber. After 24 h’ incubation, cells were fixed with 4% formaldehyde and stained with 0.5% crystal violet. Then stained cells were observed and counted under a microscope.

### Luciferase reporter gene assay

Dual-Luciferase Reporter Assay System (Promega, Madison, WI, USA; E1910) was used to assess the regulation of FOXO1 on the transcriptional activity of ARHGAP15. In brief, the promoter sequence of ARHGAP15 was cloned into pGL3-basic plasmid, which contained firefly luc2 gene, to construct the luciferase expression vector. Then the recombinant plasmids together with pRL-TK, which contained Rluc encoding renilla luciferase and was used as internal control, were co-transfected into LoVo cells. Subsequently, lentiviruses were transduced to knockdown or elevate FOXO1 expression. Firefly luciferase activity and renilla luciferase activity were measured successively to calculate the ratio.

### Xenograft mouse model and in vivo metastasis mouse model

The animal study was performed in accordance with the Guidelines for the Animal Care and Use (Shanghai Eighth People Hospital). RKO cells were transduced with ARHGAP15 expression lentivirus (pLVX-ARHGAP15) or control Vector lentivirus (pLVX-NC), and stable cell lines were established by selection with puromycin. For xenograft mouse model, 12 five-week old nude mice (Shanghai Laboratory Animal Company, Shanghai, China) were divided into two groups and subcutaneously injected with Vector or ARHGAP15-overexpressed RKO cells respectively. The maximum diameter (a) and minimum diameter (b) of tumor were measured with a vernier caliper every 3 days after tumor formation. Tumor volumes were calculated using (*a* × *b*^2^)/2. Finally, all the mice were killed and the xenograft was collected for western blotting.

For in vivo metastasis mouse model, Vector or ARHGAP15-overexpressed RKO cells (2 × 10^6^) were injected into the lateral tail vein of 5-week old nude mice (*n* = 6 per group). Eight weeks later, the mice were killed. The lungs were obtained for counting the number of the metastasis foci formation in the lung and then processed for hematoxylin and eosin staining.

### Statistical analysis

Statistical analyses were performed using the Statistical Package for the Social Sciences software version 16.0 (SPSS, Inc., Chicago, IL, USA). The correlation between ARHGAP15 expression and clinicopathological features was assessed by Fisher’s exact test. Kaplan–Meier survival cures and log-rank test were used to analyze overall survival. Comparison of differences between groups was performed by Student’s *t* test and one-way analysis of variance with Tukey’s post hoc test. Statistical significance was set at *P* < 0.05.

## Electronic supplementary material

Supplemental figure legends

supplementary figure 1

supplementary figure 2

Supplementary figure 3

Supplementary figure 4

Supplementary figure 5
