# Supplementary material for: Decreased expression of ARHGAP15 promotes the development of colorectal cancer through PTEN/AKT/FOXO1 axis
Source: Cell Death Dis. 2018 Jun 4;9(6):673. doi: 10.1038/s41419-018-0707-6 (PMC5986807; doi:10.1038/s41419-018-0707-6)
Supplement: Supplementary file 1 — Supplemental figure legends [file 41419_2018_707_MOESM1_ESM.docx]

**Figure S1. Enforced ARHGAP15 expression in CRC cells.** (A, B) mRNA (A) and protein (B) levels of ARHGAP15 in normal intestinal epithelial cells and CRC cell lines. (C-F) HT29 (C, D) and RKO (E, F) cells were transfected with pLVX-ARHGAP15 or pLVX-NC. ARHGAP15 overexpression confirmed by qRT-PCR (C, E) and western blot (D, F). Experiments were repeated three times independently. NS: no significant difference; *** P<0.001.

**Figure S2. Downregulated ARHGAP15 experssion in LoVo cells.** LoVo cells were transfected with sh-NC or sh-ARHGAP15 #1, #2 and #3. ARHGAP15 silencing confirmed by qRT-PCR (A) and western blot (B). Experiments were repeated three times independently. NS: no significant difference; *** P<0.001.

**Figure S3.** **GSEA analysis** with TCGA CORD datasets revealed that PTEN pathway was positively correlated with ARHGAP15 expression in CRC samples. NES, normalized enrichment score.

**Figure S4.** **Enforced PTEN expression in LoVo cells.** LoVo cells were transfected with pLVX-PTEN or pLVX-NC. PTEN overexpression confirmed by qRT-PCR (A) and western blot (B). Experiments were repeated three times independently. NS: no significant difference; ***P<0.001.

**Figure S5. Manipulation of FOXO1 expression in LoVo cells.** (A, B) LoVo cells were transfected with sh-NC or sh-FOXO1 #1, #2 and #3. FOXO1 silencing confirmed by qRT-PCR (A) and western blot (B). (C, D) LoVo cells were transfected with pLVX-FOXO1 or pLVX-NC. FOXO1 overexpression confirmed by qRT-PCR (A) and western blot (B). Experiments were repeated three times independently. NS: no significant difference; *** P<0.001.
