# Supplementary figures and images for: Decreased expression of ARHGAP15 promotes the development of colorectal cancer through PTEN/AKT/FOXO1 axis
Source: Cell Death Dis. 2018 Jun 4;9(6):673. doi: 10.1038/s41419-018-0707-6 (PMC5986807; doi:10.1038/s41419-018-0707-6)

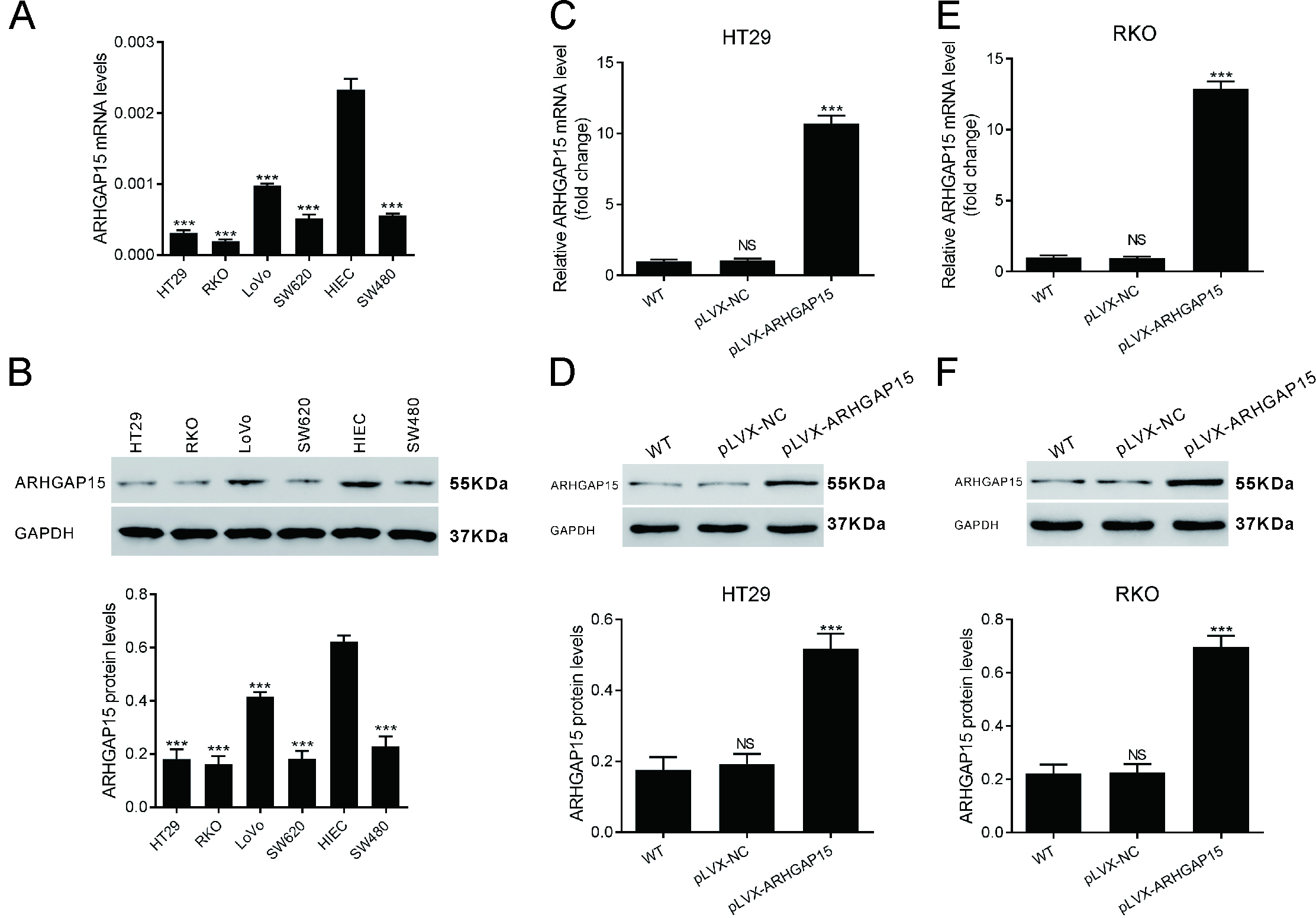

Supplement: Supplementary file 2 — supplementary figure 1 [file 41419_2018_707_MOESM2_ESM.jpg]

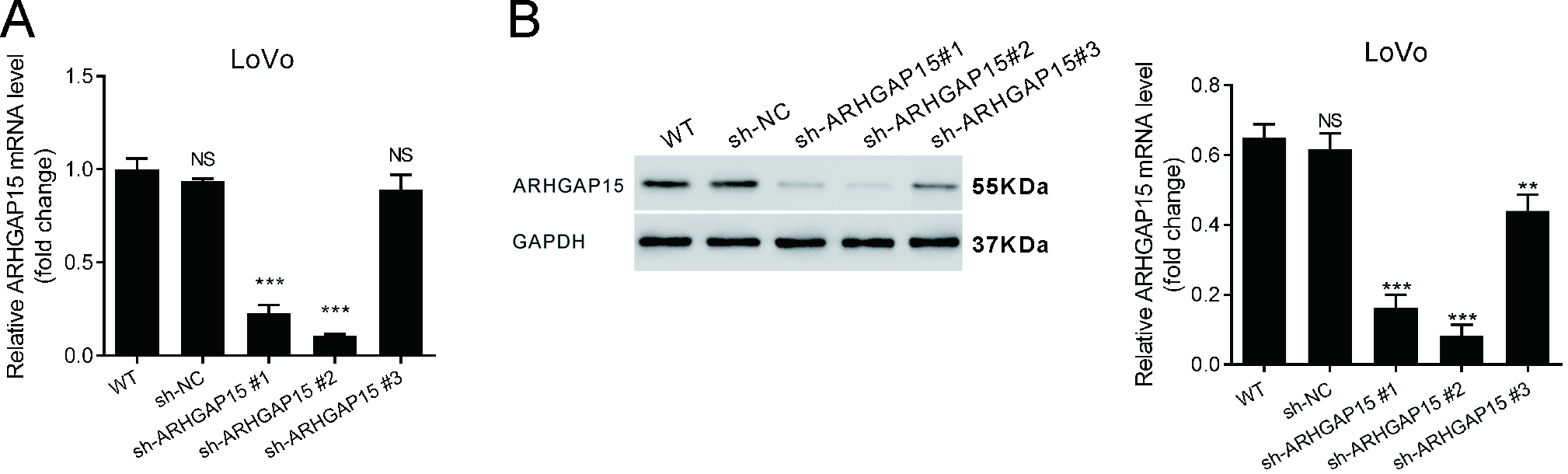

Supplement: Supplementary file 3 — supplementary figure 2 [file 41419_2018_707_MOESM3_ESM.jpg]

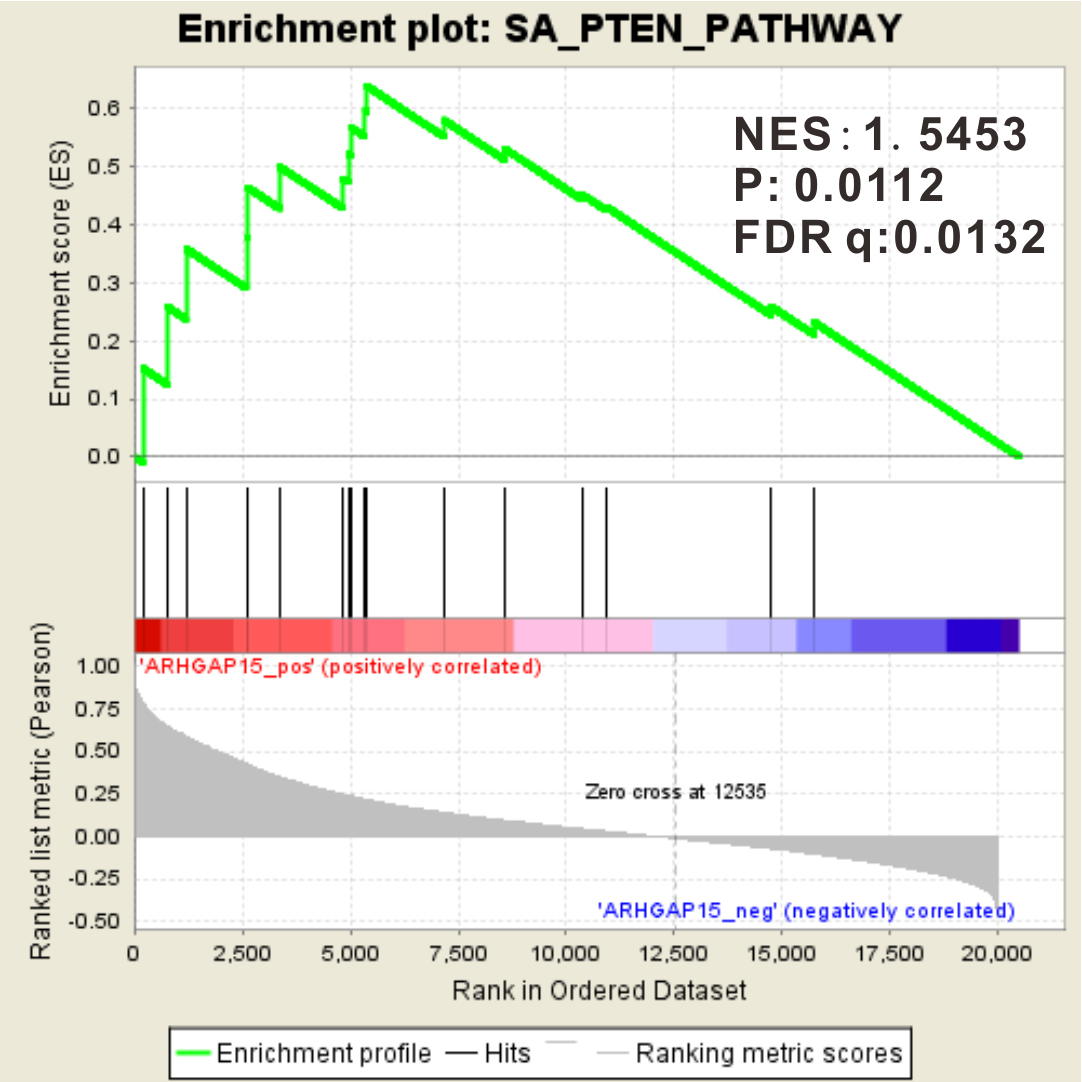

Supplement: Supplementary file 4 — Supplementary figure 3 [file 41419_2018_707_MOESM4_ESM.jpg]

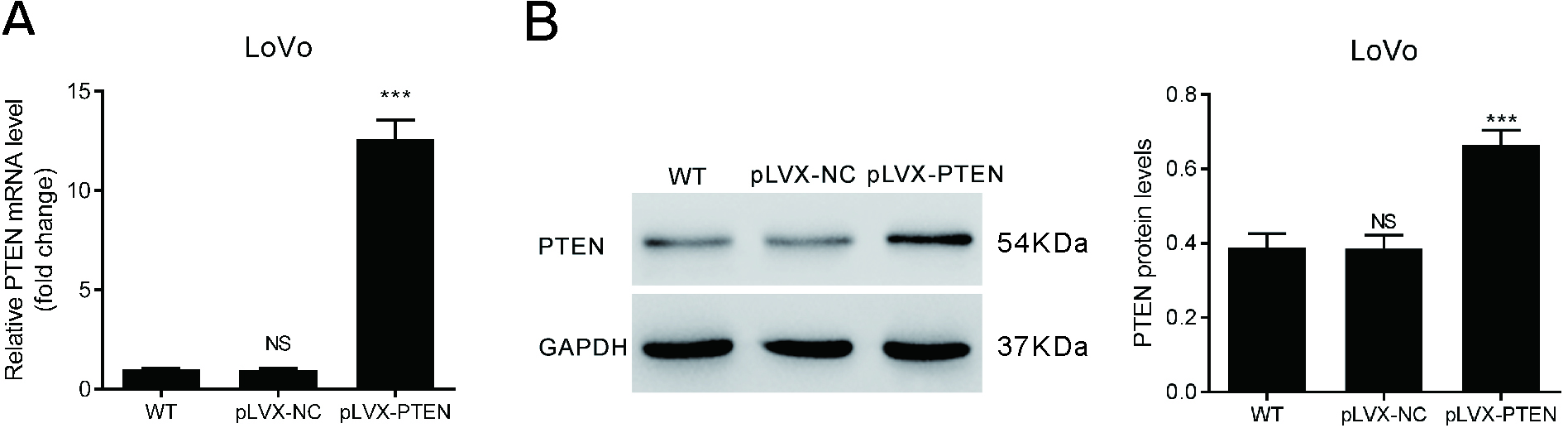

Supplement: Supplementary file 5 — Supplementary figure 4 [file 41419_2018_707_MOESM5_ESM.jpg]

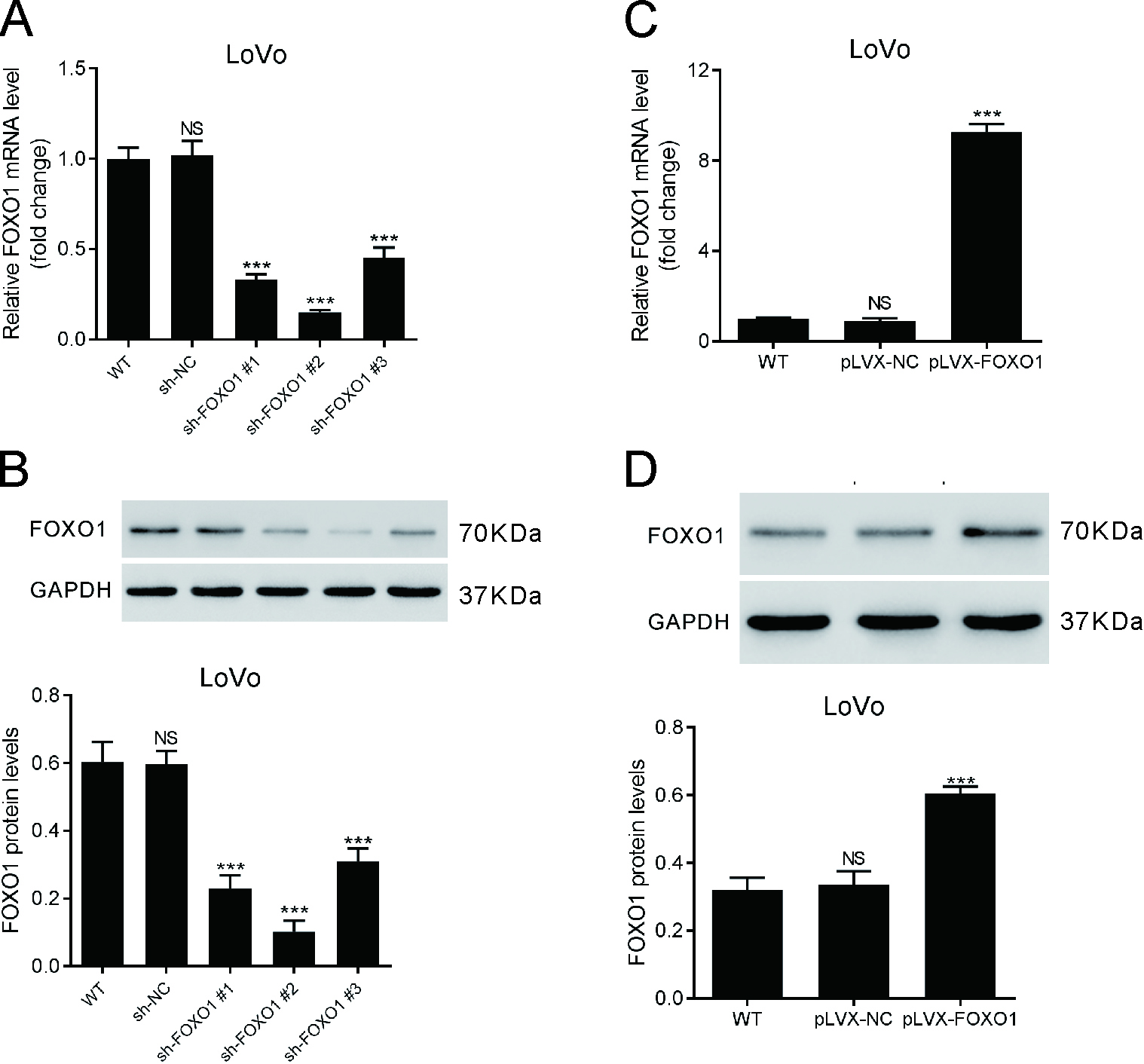

Supplement: Supplementary file 6 — Supplementary figure 5 [file 41419_2018_707_MOESM6_ESM.jpg]
